# Low-temperature-grown continuous graphene films from benzene by chemical vapor deposition at ambient pressure

**DOI:** 10.1038/srep17955

**Published:** 2015-12-10

**Authors:** Jisu Jang, Myungwoo Son, Sunki Chung, Kihyeun Kim, Chunhum Cho, Byoung Hun Lee, Moon-Ho Ham

**Affiliations:** 1 Center for Emerging Electronic Devices and Systems, Department of Nanobio Materials and Electronics, School of Materials Science and Engineering, Gwangju Institute of Science & Technology, 123 Cheomdangwagi-ro, Buk-gu, Gwangju 61005, Republic of Korea

## Abstract

There is significant interest in synthesizing large-area graphene films at low temperatures by chemical vapor deposition (CVD) for nanoelectronic and flexible device applications. However, to date, low-temperature CVD methods have suffered from lower surface coverage because micro-sized graphene flakes are produced. Here, we demonstrate a modified CVD technique for the production of large-area, continuous monolayer graphene films from benzene on Cu at 100–300 °C at ambient pressure. In this method, we extended the graphene growth step in the absence of residual oxidizing species by introducing pumping and purging cycles prior to growth. This led to continuous monolayer graphene films with full surface coverage and excellent quality, which were comparable to those achieved with high-temperature CVD; for example, the surface coverage, transmittance, and carrier mobilities of the graphene grown at 300 °C were 100%, 97.6%, and 1,900–2,500 cm^2^ V^−1^ s^−1^, respectively. In addition, the growth temperature was substantially reduced to as low as 100 °C, which is the lowest temperature reported to date for pristine graphene produced by CVD. Our modified CVD method is expected to allow the direct growth of graphene in device manufacturing processes for practical applications while keeping underlying devices intact.

Graphene, a two-dimensional monolayer of *sp*^2^-hybridized carbon atoms arranged in a honeycomb lattice, has received significant interest due to its extraordinary electronic, optical, mechanical, and chemical properties^1^,^2^,^3^,^4^. Since its first isolation by mechanical exfoliation from graphite in 2004, large-scale production of graphene films has been an urgent issue for realizing practical graphene-based applications^5^. Several approaches, including chemical exfoliation of graphite^6^,^7^, annealing of SiC substrates^8^,^9^, catalytic chemical vapor deposition (CVD) with carbon feedstock^10^,^11^,^12^,^13^,^14^, and pyrolysis of polymers^15^,^16^ have been attempted to prepare graphene. Among them, the CVD process is favored for the synthesis of large-area, high-quality graphene, at least to date, and meets the requirements for industrialization^10^,^11^,^12^,^13^,^14^. A current CVD route typically requires high temperatures of 800–1,000 °C for pyrolytic cracking of hydrocarbon gases^10^,^11^,^12^,^13^,^14^. However, the high-temperature process requires expensive and precise equipment and makes the direct deposition of graphene in electronic device manufacturing processes infeasible due to the severe physical damage to substrates (*e.g.*, metals, semiconductors, and the junctions between them) underneath the graphene. It is therefore indispensable to synthesize graphene at reduced growth temperatures, permitting large-area fabrication with relatively low cost. Several groups have demonstrated that a drastic reduction in growth temperature can be achieved by using plasma-enhanced CVD (PECVD), where plasma generates reactive species^17^,^18^, or thermal CVD with aromatic molecules as carbon sources^19^,^20^,^21^. Recent studies demonstrated that the growth temperature could be decreased to as low as 380–500 °C by using PECVD^17^,^18^, but the PECVD can cause the collateral damage on graphene surface by energetic plasma ions. Using benzene enabled the synthesis of graphene at lower temperatures (~300 °C) because of its low activation energy, but it did not lead to large-area, continuous graphene films^19^,^21^. Although in some cases low-temperature graphene growth has been demonstrated, achieving continuous graphene films at low temperatures has remained a challenge, particularly below 300 °C, which would keep underlying substrates intact.

Additionally, the typical graphene growth routes are mostly performed in low-pressure conditions in order to achieve a high degree of graphene crystallinity. Although the vacuum CVD process has been widely used for graphene growth, atmospheric pressure CVD (APCVD) growth of graphene of appreciable quality is desirable in order to lower the manufacturing cost and increase throughput. Moreover, severe evaporation of metal catalysts in low-pressure conditions, which may lead to deterioration of the graphene quality, can be significantly suppressed by increasing the working pressure. However, oxygen and water molecules in ambient air should be avoided to obtain high-quality graphene. In this study, we developed a modified APCVD route to synthesize graphene on Cu foils, which is called the “oxygen-free APCVD” process. In this method, we extended the graphene growth step in the absence of residual oxidizing species that may influence the formation of amorphous and oxidized carbon layers. Using this method with benzene as a carbon feedstock, large-area, continuous graphene sheets with excellent quality were synthesized on Cu foils at 300 °C. The growth temperature was substantially reduced to as low as 100 °C, which is the lowest temperature reported to date for pristine graphene produced by CVD.

## Results

### Low-temperature synthesis of continuous graphene films by oxygen-free APCVD

**Figure 1:**
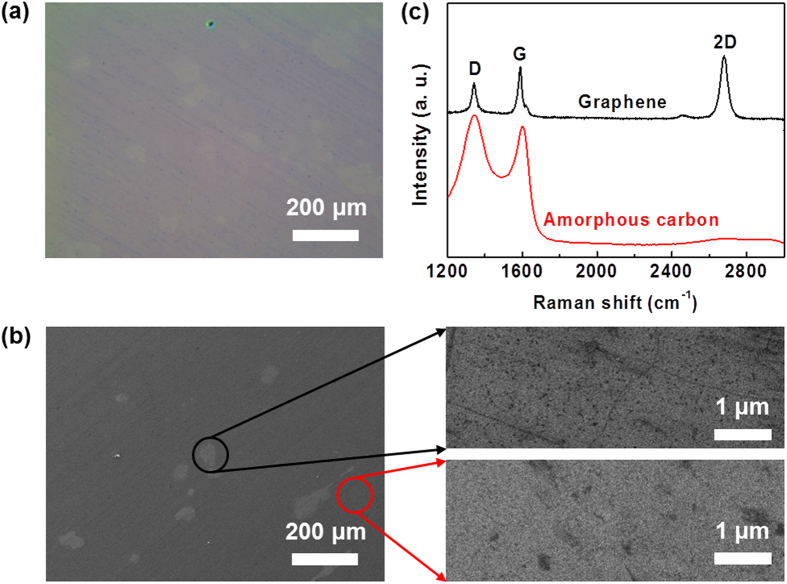
(**a**) OM image, (**b**) SEM images, and (**c**) Raman spectra of carbon film grown by normal APCVD at 300 °C. The carbon film consists of graphene flakes (upper right SEM image of (**b**) and upper Raman spectrum of (**c**)) and unwanted amorphous and oxidized carbon regions (lower right SEM image of (**b**) and lower Raman spectrum of (**c**)).

**Figure 2:**
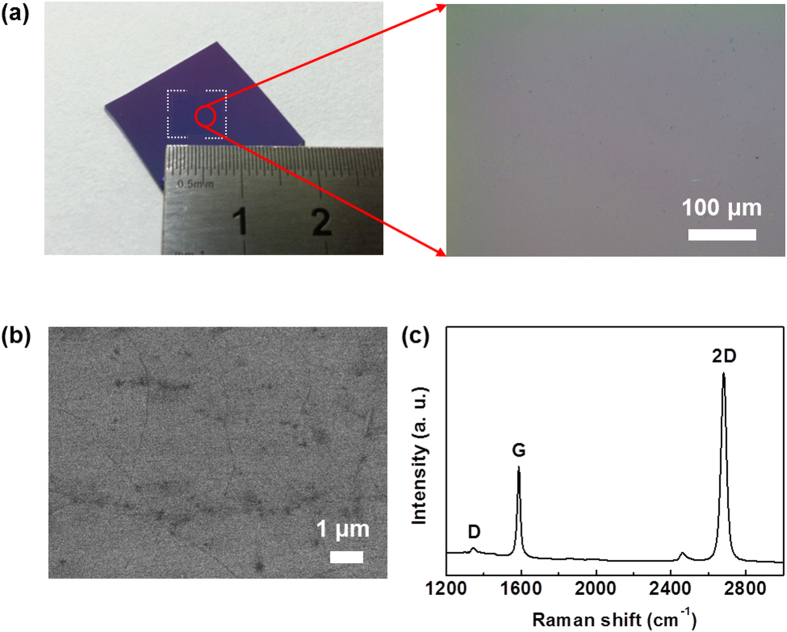
(**a**) Photograph and OM image, (**b**) SEM image, and (**c**) Raman spectrum of continuous monolayer graphene film grown by oxygen-free APCVD at 300 °C for 5 min.

Growth on Cu foils from benzene by “normal APCVD” at 300 °C produced a carbon layer with a low coverage of graphene, which predominantly consisted of unwanted amorphous and oxidized carbon regions (Fig. 1). In a “normal APCVD” process, the gas flow rates of active components are typically higher than in a low-pressure CVD (LPCVD) process, and thus gas phase reactions, including the reactions with oxidizing species, can occur in the bulk gas flow^22^,^23^. Therefore, particulates may be deposited on the surface of Cu foils, resulting in the inhomogeneous nucleation of carbon atoms and the introduction of multiple and/or disordered carbon atoms^24^. In addition, because the “vacuum-free” APCVD reactor is kept in ambient air for most of the growth time and is exposed to trace amounts of oxidizing contaminants, the inevitable presence of residual oxidizing species during the growth process leads to the oxidative etching of graphene, which introduces defects in the graphene^25^,^26^,^27^,^28^. To solve this problem, we extended the graphene growth step in the absence of oxidizing contaminants in a process called the “oxygen-free APCVD”. Prior to graphene growth, we conducted several cycles of pumping and purging. This approach can effectively remove residual oxidizing impurities from the APCVD system during the growth process (Supplementary Fig. S1). Figure 2a,b show a photograph and optical microscopy (OM) and scanning electron microscopy (SEM) images of a centimeter-scale graphene film grown at 300 °C by “oxygen-free APCVD” and transferred onto a SiO_2_/Si substrate. The OM and SEM images show the same contrast over the entire film area, in contrast to the film grown by APCVD. This reveals the formation of a continuous graphene film. Moreover, the Raman measurement indicates a monolayer graphene with a low defect density (Fig. 2c). These results are similar to those for films produced from methane gas at ~1,000 °C by LPCVD^11^, but in contrast with those produced from benzene at 300 °C by LPCVD^19^,^21^. This implies that the growth mechanism of our oxygen-free APCVD method differs from that of the LPCVD method.

**Figure 3:**
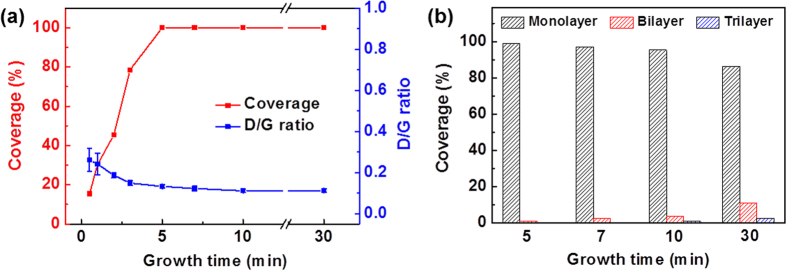
(**a**) Surface coverage and intensity ratio of D band to G band (D/G ratio) in Raman spectra of graphene films grown by oxygen-free APCVD at 300 °C for different times (30 sec to 30 min). Surface coverage was estimated from OM images of Supplementary Fig. S2, and the D/G ratio was obtained from Raman data of Supplementary Figs S3 and S4. (**b**) Surface coverage distribution of graphene films grown by oxygen-free APCVD at 300 °C for different times (5–30 min). Surface coverage distribution was estimated from Raman mapping data of Supplementary Fig. S4.

In the oxygen-free APCVD process, the introduction of pumping and purging cycles enabled the continuous film growth of graphene at 300 °C. We then tried to grow the graphene at 300 °C for different times (30 sec to 30 min). For the growth time of 30 sec, the defective graphene flakes were synthesized on a Cu foil (Fig. 3a, and Supplementary Figs S2 and S3). As the growth time increased, the surface coverage increased by the lateral domain growth (Fig. 3a and Supplementary Fig. S2). In Raman spectra, the 2D peaks became stronger and *I*_D/G_ decreased (Fig. 3a and Supplementary Fig. S3), revealing that the quality of graphene is improved with growth time due to a decrease in defects such as edges and dangling bonds^18^,^29^. The graphene grown for 5 min showed 100% surface coverage and uniform *I*_2D/G_ (>1.8), which indicates the formation of a uniform, continuous graphene film with the monolayer coverage of >99% by merging adjacent graphene domains (Fig. 3 and Supplementary Figs S2–S4). For longer growth times, bilayer and trilayer graphene flakes started to be formed on the monolayer films (Fig. 3 and Supplementary Figs S2–S4).

**Figure 4:**
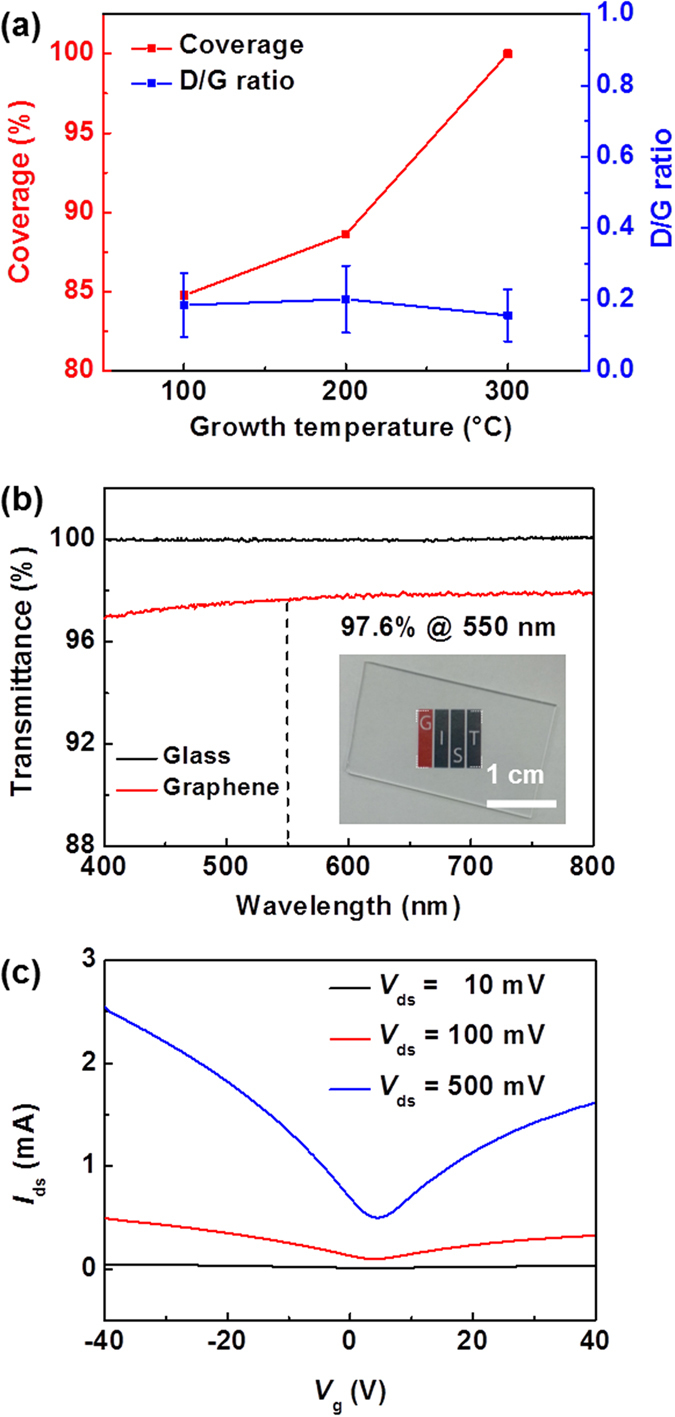
(**a**) Surface coverage and D/G ratio in Raman spectra of graphene films grown by oxygen-free APCVD at different temperatures (100–300 °C). Surface coverage was estimated from OM images of Supplementary Fig. S1, and the D/G ratio was obtained from Raman mapping data of Supplementary Figs S2b-S2d. (**b**) Optical transmittance of graphene film grown by oxygen-free APCVD at 300 °C on a glass substrate. The inset shows a photograph of graphene film on a glass substrate. (**c**) *I*_ds_−*V*_g_ characteristics of graphene FET recorded at various *V*_ds_ at room temperature in vacuum.

Further, we tried to grow the graphene at lower temperatures between room temperature and 200 °C. A previous study theoretically predicted that graphene could be grown even at 200 °C by estimating the dehydrogenation rate based on first-principle calculations^21^. As the growth temperature decreased to 100 °C, graphene films were synthesized, but the surface coverage decreased owing to a decrease in the catalytic activity of the system, which showed a nearly linear dependence on temperature (Fig. 4a and Supplementary Fig. S5) and is consistent with the previous report^30^. However, at room temperature, graphene was not synthesized at all (Supplementary Figs S5 and S6). It is believed that a minimal thermal energy is required for graphene growth involving the adsorption and dehydrogenation of benzene molecules and C-C bond formation, although the origin of the graphene growth at 100 °C still remains unclear. In Raman spectra, the full width at half maximum (FWHM) of the 2D bands for all the graphene films was ~35 cm^−1^ (Supplementary Fig. S6a), indicating monolayer graphene. This feature is similar to those for CH_4_-derived monolayer graphene^11^. Although D bands due to defects and/or populous domain boundaries exist, as confirmed by the SEM images (Fig. 2b)^31^, their intensities are quite low (Figs 2c and 4a, and Supplementary Fig. S6). This indicates the formation of high-quality graphene regardless of growth temperature. Importantly, a comparison of Raman spectra and OM images for the films grown by oxygen-free APCVD and normal APCVD reveals that the introduction of pumping and purging cycles suppresses the formation of amorphous and oxidized carbon layers, improves the quality of graphene, and facilitates the growth of continuous graphene because of the absence of oxidizing impurities (Figs 1 and 2). Therefore, the removal of such oxidizing species is imperative for the production of large-area and high-quality, continuous graphene films while permitting growth at low temperatures. Additionally, the optical transmittance at 550 nm for the graphene grown at 300 °C and transferred onto a glass substrate was 97.6% (Fig. 4b). Considering 2.3% absorption of incident white light in a graphene layer^32^, this value indicates predominantly monolayer graphene, which is in good agreement with the Raman and OM results of the same graphene and comparable to those of LPCVD-grown monolayer graphene^11^,^33^.

### Electrical properties of graphene films grown by oxygen-free APCVD

The quality of our graphene film grown by oxygen-free APCVD was further tested by electrical transport measurements in a field-effect transistor (FET) configuration, which was fabricated on a 300 nm-thick SiO_2_/Si substrate with Ti/Au as source/drain electrodes and heavily *p*-doped Si as a back gate. Figure 4c shows typical transfer characteristics of the graphene device grown at 300 °C, measured at room temperature in vacuum. The electron and hole field-effect mobility (*μ*_FE_) of the device were 1,900 and 2,500 cm^2^ V^−1^ s^−1^, respectively^14^. Additionally, the sheet resistance of the graphene was ~1,000 Ω/sq. The carrier mobilities and sheet resistance are much better than those of low-temperature-grown graphene^20^,^34^, but comparable to those of CH_4_-derived monolayer graphene at ~1,000 °C by LPCVD^11^,^20^,^35^.

### Growth mechanism of continuous graphene films

**Figure 5:**
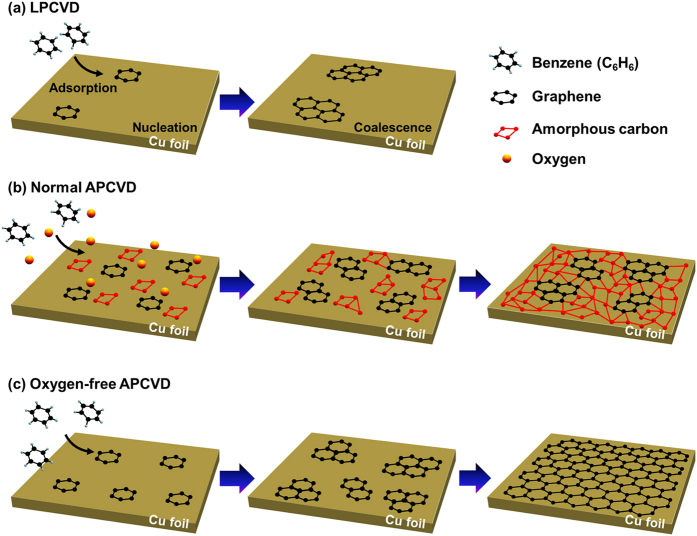
Schematic diagrams showing the growth mechanisms of graphene on a Cu surface from benzene by (**a**) LPCVD, (**b**) normal APCVD, and (**c**) oxygen-free APCVD.

The graphene growth from aromatic benzene molecules involves the following steps: (1) benzene molecules are introduced into the furnace by bubbling liquid benzene with argon and hydrogen as the carrier gases at ambient pressure; (2) the carbon sources then undergo adsorption on Cu surfaces and catalytic dehydrogenation at low temperatures to form highly reactive hexahydric ring species, leading to the formation of graphene nuclei; (3) continual growth of graphene takes place when hexahydric ring-containing molecules are incorporated into the edges of graphene nuclei that are chemically active (Fig. 5). In previous studies, the low-temperature growth of graphene from benzene suffered from much lower film coverage, and only graphene flakes with a size limited to several micrometers were formed when LPCVD methods were used^19^,^21^. Although the high adsorption energy of benzene due to strong London dispersion force, which helps to prevent the adsorbed molecules from desorption and facilitate their dehydrogenation, enables the low-temperature growth of graphene^21^, the probability of adsorption of benzene molecules onto the Cu surface is reduced at low pressures (Fig. 5a). This leads to reduced probabilities of both dehydrogenation and C-C bond formation, thus limiting the domain growth of graphene^21^,^24^,^36^. However, nucleation occurs sufficiently at ambient pressure. APCVD growth leads to continuous but defective carbon films with unwanted amorphous and oxidized carbon regions because of the existence of oxygen species during growth (Fig. 5b), whereas, in the oxygen-free APCVD process, continuous graphene films are synthesized with full surface coverage due to the absence of residual oxidizing species (Fig. 5c). Furthermore, oxidative etching of graphene rarely occurs in the oxygen-free APCVD process, resulting in a sufficiently low defect density (Fig. 2c)^26^,^27^,^28^. Therefore, the growth of continuous graphene films by our oxygen-free APCVD method could depend on a delicate balance between adsorption, dehydrogenation, C-C bond formation, and residual desorption in a residual oxygen-free environment. It has been reported by by Ruoff *et al.* that oxygen influences graphene nucleation and reduces the nucleation density^37^. They showed the formation of centimeter-scale single-crystal graphene domains by oxygen-controlled approach. Through the precise oxygen control in our modified APCVD method, the quality and domain size of the continuous graphene films could be improved.

## Discussion

We developed a modified CVD route, called the oxygen-free APCVD process, to synthesize large-area, continuous monolayer graphene films from benzene on Cu at as low as 300 °C at ambient pressure. The oxygen-free APCVD process introduces a step consisting of pumping and purging cycles prior to graphene growth, which leads to the absence of oxidizing impurities during growth and thus to the formation of uniform and continuous monolayer graphene films of improved quality. The graphene grown at 300 °C had properties comparable to those of 1,000 °C-grown graphene, with surface coverage of 100%, optical transmittance of 97.6%, and field-effect mobilities of 1,900–2,500 cm^2^ V^−1^ s^−1^. Even when the growth temperature was substantially lowered to 100 °C, graphene films were synthesized, but with reduced surface coverage. This demonstration is a significant step toward the direct growth of graphene in device manufacturing processes for practical applications such as CMOS back-end interconnects and flexible devices.

## Methods

### Synthesis of graphene by oxygen-free APCVD

Cu foil (25 μm, Alfa Aesar) was used as a catalytic substrate to grow graphene. The Cu foil was first cleaned with 18% HCl, deionized water, acetone, and isopropyl alcohol and subsequently dried with nitrogen. Then, it was loaded into the hot center of a quartz tube. Prior to graphene growth, the quartz tube was pumped down to ~10^−4^ Torr and then purged with argon. This process was repeated five times to flush the air contained in the quartz tube (residual oxygen concentration of 0%). After a mixture of hydrogen and argon (100 and 400 sccm, respectively) was supplied into the quartz tube, the pump was switched off, and the quartz tube was kept at ambient pressure. To increase the Cu grain size and ensure the removal of native oxide and a smooth Cu surface, the Cu foil was annealed at 1,000 °C for 30 min under a hydrogen and argon atmosphere and was subsequently rapidly cooled down to the desired growth temperature (25–300 °C). Once the desired temperature was reached, the flow rate of hydrogen gas was changed to 20 sccm without flowing argon (purging gas) while the carbon source was introduced by bubbling liquid benzene (≥99.9%, Sigma-Aldrich) at room temperature. The carbon source was supplied to the quartz tube with 5 sccm of argon flow (carrier gas) for 5 min, producing a monolayer graphene. The graphene growth was completed by stopping the supply of benzene, and the furnace was subsequently cooled to room temperature while hydrogen and argon (40 and 400 sccm, respectively) continued flowing without supplying benzene. For comparison, graphene was synthesized at 300 °C by APCVD (residual oxygen concentration of 0.2%) without the introduction of the pumping and purging step. To transfer the graphene onto a heavily *p*-doped Si substrate with a thermally grown 300 nm-thick SiO_2_ layer or a glass substrate, poly(methyl methacrylate) (PMMA, M_w_ = 950,000 g/mol, dissolved in 4% anisole, MicroChem) was spin-coated on the graphene/Cu, which was baked at 60 °C for 5 min to remove the solvent from the polymer^14^,^38^. The PMMA/graphene/Cu was subsequently floated on standard Cu etchant (CE-100, Transene Co., Inc.) to wet-etch away the Cu foil. The PMMA/graphene was rinsed with deionized water several times, and then transferred onto the substrate, followed by drying in air. Finally, the PMMA was dissolved by soaking the sample in acetone, thus leaving only the graphene film on the substrate.

### Device fabrication

Graphene FETs were fabricated using standard photolithography techniques. The substrate was a heavily *p*-doped Si substrate with a thermally grown 300 nm-thick SiO_2_ layer, which were used as the bottom gate and gate dielectric, respectively. After transferring the graphene sheet onto the substrate, source and drain electrodes were deposited using photolithography, followed by electron-beam evaporation of Ti/Au. Then, a second photolithography and an oxygen plasma treatment were performed to pattern the graphene channel. Typical channel width and length were 8 and 40 μm, respectively.

### Characterization

The surface morphology of graphene was visualized by optical microscopy (BX51, Olympus) and field-emission scanning electron microscopy (FESEM, JSM-7500F, JEOL). Raman spectroscopy (HR-320, Horiba Jovin-Yvon) with a laser excitation wavelength of 532 nm was used to characterize the thickness, quality, and uniformity of the graphene films. Optical transmittance was measured using an UV-vis-nIR spectrometer (Lambda 900, Perkinelmer) to examine the thickness and uniformity of the graphene transferred onto a glass substrate. The electrical properties of the graphene were studied in vacuum at room temperature using a semiconductor parameter analyzer (E5270B, Agilent Technologies) and a four-point probe measurement system (CMT-SR 2000, Changmin Tech.).

## Additional Information

**How to cite this article**: Jang, J. *et al.* Low-temperature-grown continuous graphene films from benzene by chemical vapor deposition at ambient pressure. *Sci. Rep.* **5**, 17955; doi: 10.1038/srep17955 (2015).

## Supplementary Material

Supplementary Information
