# Supplementary material for: Low-temperature-grown continuous graphene films from benzene by chemical vapor deposition at ambient pressure
Source: Sci Rep. 2015 Dec 10;5:17955. doi: 10.1038/srep17955 (PMC4674705; doi:10.1038/srep17955)
Supplement: Supplementary Information [file srep17955-s1.pdf]

Supplementary Information for

Low-temperature-grown continuous graphene films from benzene by chemical vapor deposition at ambient pressure

Jisu Jang†, Myungwoo Son†, Sunki Chung, Kihyeun Kim, Chunhum Cho, Byoung Hun Lee and Moon-Ho Ham*

Center for Emerging Electronic Devices and Systems, Department of Nanobio Materials and Electronics, School of Materials Science and Engineering, Gwangju Institute of Science & Technology, 123 Cheomdangwagi-ro, Buk-gu, Gwangju 61005, Republic of Korea

*mhham@gist.ac.kr

†These authors contributed equally to this work.

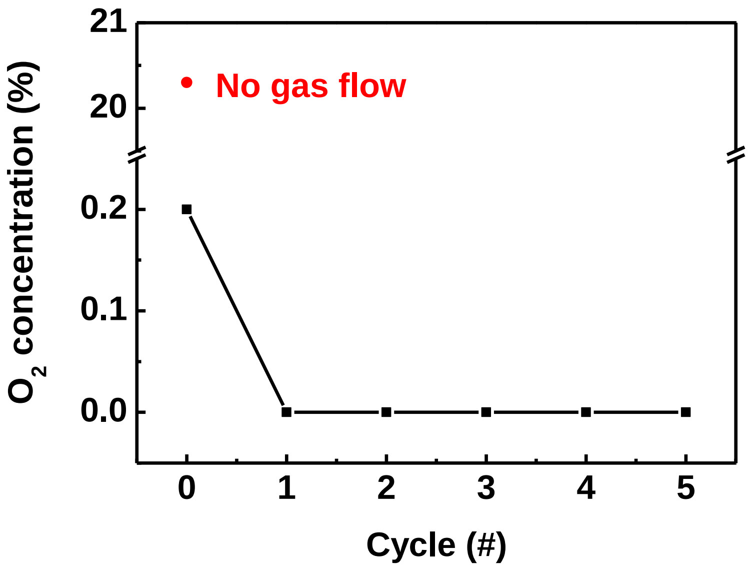

Figure S1. O_2 concentration in a CVD chamber with repeated pumping and purging cycles. The initial O_2 concentration was around 20.3%. In the CVD chamber with argon gas flow, the concentration of the residual oxygen impurities is 0.2% (2,000 ppm). When the pumping and purging processes are introduced, the concentration of the residual oxygen impurities is reduced to 0%.

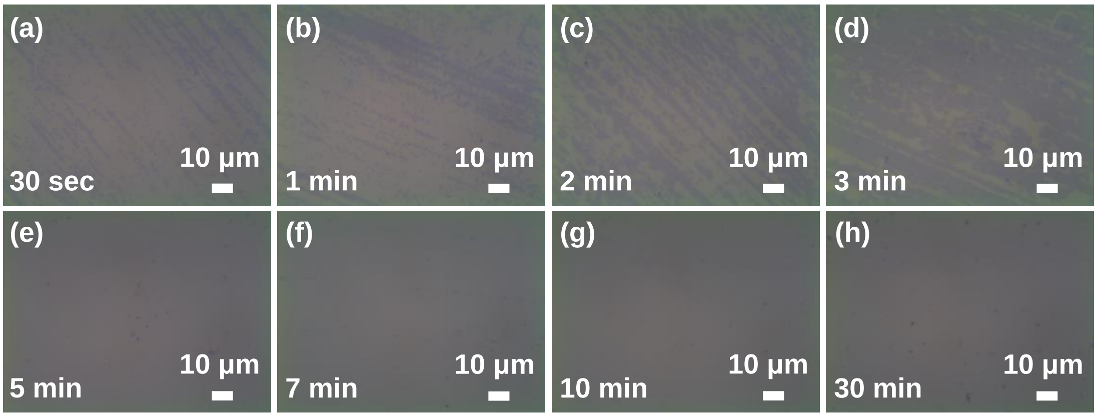

Figure S2. OM images of graphene films grown by oxygen-free APCVD at 300 ℃ for different times (30 sec to 30 min).

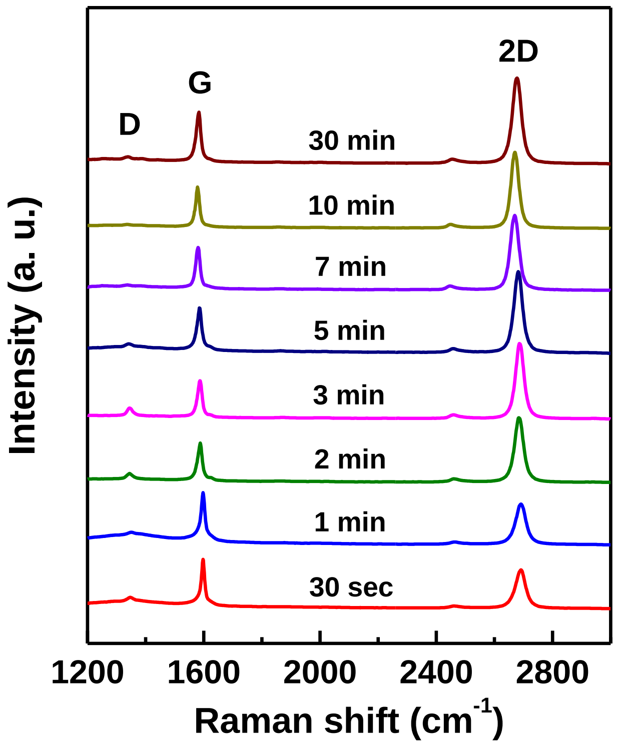

Figure S3. Raman spectra of graphene films grown by oxygen-free APCVD at 300 °C for different times (30 sec to 30 min).

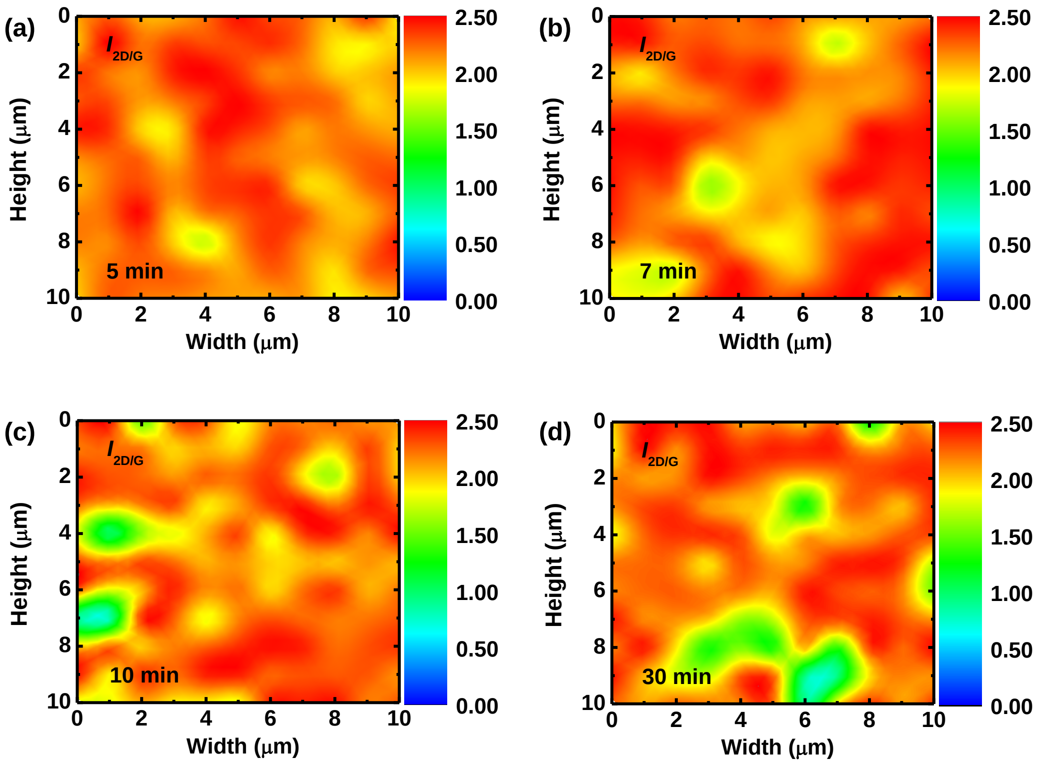

Figure S4. Raman mapping images of graphene films grown by oxygen-free APCVD at 300 °C for different times: (a) 5 min, (b) 7 min, (c) 10 min, and (d) 30 min.

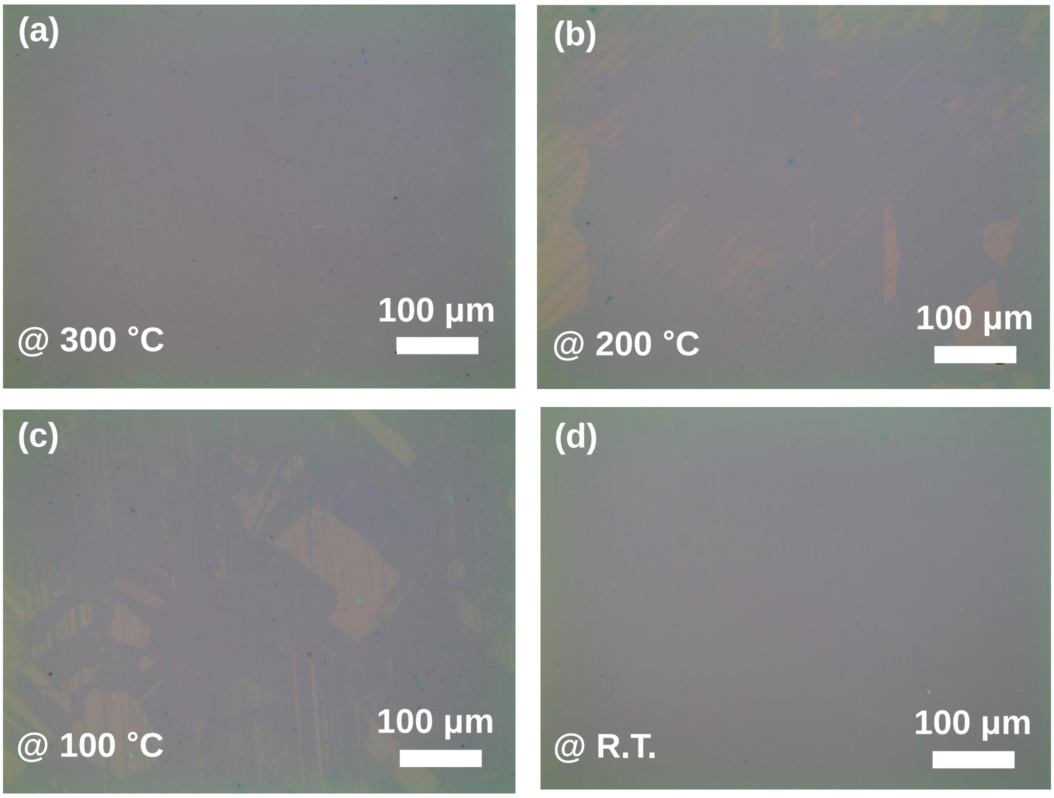

Figure S5. OM images of graphene films grown by oxygen-free APCVD at different temperatures: (a) 300 ℃, (b) 200 ℃, (c) 100 ℃, and (d) room temperature. Graphene was not synthesized at room temperature.

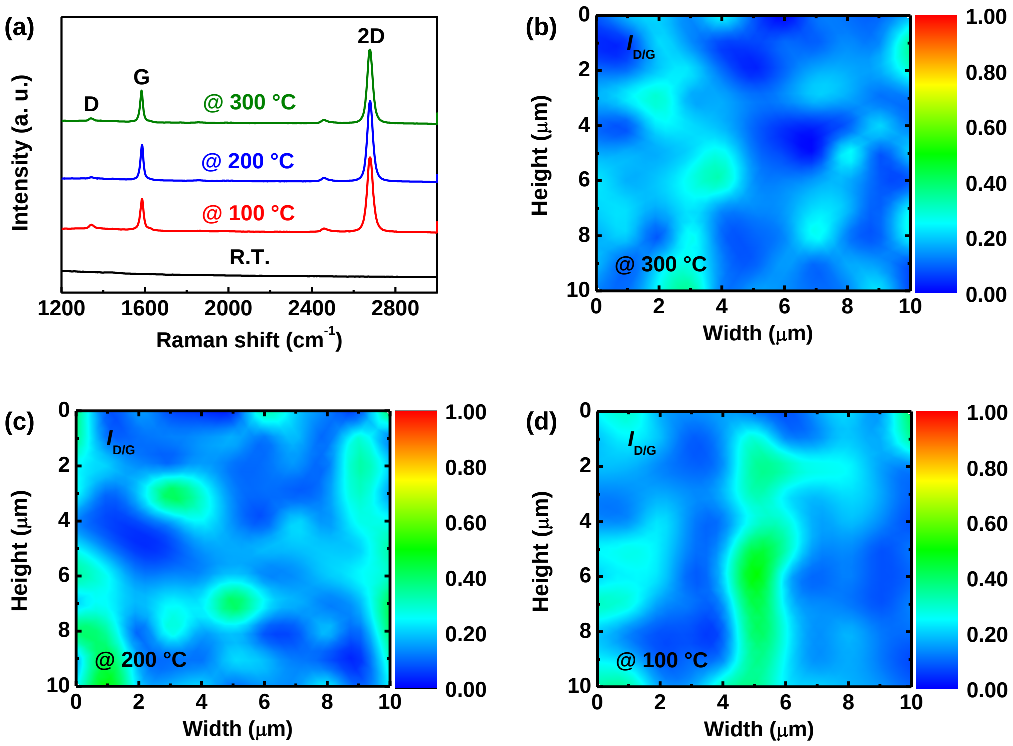

Figure S6. (a) Raman spectra of graphene films grown by oxygen-free APCVD at different temperatures. Graphene was not synthesized at room temperature. Raman mapping images of graphene films grown by oxygen-free APCVD at different temperatures: (a) 300 °C, (b) 200 °C, and (c) 100 °C.
